# Premature aging is associated with higher levels of 8‐oxoguanine and increased DNA damage in the Polg mutator mouse

**DOI:** 10.1111/acel.13669

**Published:** 2022-08-22

**Authors:** Tenghui Yu, Jesse Slone, Wensheng Liu, Ryan Barnes, Patricia L. Opresko, Landon Wark, Sabine Mai, Steve Horvath, Taosheng Huang

**Affiliations:** ^1^ Department of Pediatrics University at Buffalo Buffalo New York USA; ^2^ Human Aging Research Institute, School of Life Science Nanchang University Nanchang China; ^3^ Division of Human Genetics Cincinnati Children's Hospital Medical Center Cincinnati Ohio USA; ^4^ Department of Environmental and Occupational Health University of Pittsburgh Graduate School of Public Health, and UPMC Hillman Cancer Center Pittsburgh Pennsylvania USA; ^5^ CancerCare Manitoba Research Institute, The Genomic Center for Cancer Research & Diagnosis University of Manitoba Winnipeg Manitoba Canada; ^6^ Human Genetics, David Geffen School of Medicine University of California Los Angeles Los Angeles California USA

**Keywords:** 8‐oxoguanine, aging, mitochondria, oxidative stress, telomeres

## Abstract

Mitochondrial dysfunction plays an important role in the aging process. However, the mechanism by which this dysfunction causes aging is not fully understood. The accumulation of mutations in the mitochondrial genome (or “mtDNA”) has been proposed as a contributor. One compelling piece of evidence in support of this hypothesis comes from the *Polg* ^*D257A/D257A*^ mutator mouse (*Polg* ^*mut/mut*^). These mice express an error‐prone mitochondrial DNA polymerase that results in the accumulation of mtDNA mutations, accelerated aging, and premature death. In this paper, we have used the *Polg* ^*mut/mut*^ model to investigate whether the age‐related biological effects observed in these mice are triggered by oxidative damage to the DNA that compromises the integrity of the genome. Our results show that mutator mouse has significantly higher levels of 8‐oxoguanine (8‐oxoGua) that are correlated with increased nuclear DNA (nDNA) strand breakage and oxidative nDNA damage, shorter average telomere length, and reduced mtDNA integrity. Based on these results, we propose a model whereby the increased level of reactive oxygen species (ROS) associated with the accumulation of mtDNA mutations in *Polg* ^*mut/mut*^ mice results in higher levels of 8‐oxoGua, which in turn lead to compromised DNA integrity and accelerated aging via increased DNA fragmentation and telomere shortening. These results suggest that mitochondrial play a central role in aging and may guide future research to develop potential therapeutics for mitigating aging process.

## Graphical abstract

The increased mtDNA mutation rate in the Polg mutator mouse has been previously shown to be associated with an accelerated aging phenotype, likely due to increased oxidative stress. This study provides evidence in support of this oxidative stress model by showing that the Polg mutation is also associated with increased levels of 8‐oxoGua as well as telomere shortening and other forms of DNA damage.

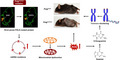

**Abbreviations**
8‐oxoGua: 8‐oxoguanine
8‐oxodG: 8‐oxo‐2’‐deoxyguanosine
DNAm: DNA methylation age
DSB: Double‐strand break
Fpg: Formamidopyrimidine DNA glycosylase
MEFs: Mouse embryonic fibroblasts
MELAS: Mitochondrial myopathy, encephalopathy, lactic acidosis, and stroke‐like episodes
MERRF: Myoclonic epilepsy with ragged red fibers
mtDNA: Mitochondrial DNA
mtDNAcn: Mitochondrial DNA copy number
nDNA: Nuclear DNA
PCR: Polymerase chain reaction
PNA: Peptide nucleic acid
POLG: DNA Polymerase gamma
Q‐FISH: Quantitative fluorescence in situ hybridization
ROS: Reactive oxygen species
SSB: Single‐strand break
TA: Telomere aggregate
UPLC‐MS/MS: Ultraperformance liquid chromatography tandem mass spectrometry

## 1 INTRODUCTION

The world population continues to undergo sustained changes due to an increase in life expectancy. In 2020, the global population of individuals 65 years of age and older was 727 million, and that proportion of the population is expected to increase from 9.3% in 2020 to 16.0% in 2050 (Kamiya et al., 2020). In such a rapidly aging world, research on the fundamental mechanisms of aging and potential treatments for age‐related maladies is likely to become an increasingly important public health priority. At the biological level, aging is caused by the long‐term accumulation of various toxic molecules and cellular damage. Several candidate hallmarks have been proposed to cause aging, including genomic instability, telomere attrition, epigenetic alterations, loss of proteostasis, deregulated nutrient sensing, mitochondrial dysfunction, cellular senescence, stem cell exhaustion, and altered intercellular communication (Aunan et al., 2016; Boonekamp et al., 2013; Caldeira da Silva et al., 2008; Harries et al., 2011; López‐Otín et al., 2013; Mesquita et al., 2010; Wallace, 2018; Wilkinson et al., 2012). The genomic instability, telomere attrition, epigenetic alterations, and loss of proteostasis are generally considered to be the primary causes of cellular damage, while deregulated nutrient sensing, mitochondrial dysfunction, and cellular senescence are considered to be antagonistic responses to that damage. On the contrary, stem cell exhaustion and altered intercellular communication are considered to be the ultimate causes of age‐related functional decline (López‐Otín et al., 2013). Together, these hallmarks influence and regulate one another and interact with the phylogenetic history of a given species to determine its aging process and longevity (López‐Otín et al., 2013; van der Rijt et al., 2020).

The mitochondrial organelle is the primary source of energy in the eukaryotic cell, providing over 90% of the ATP utilized by the cell (Pizzorno, 2014). Mitochondria are thought to play a role in aging and participate in the aging process through various pathways (Balaban et al., 2005; Seo et al., 2010; van der Rijt et al., 2020). In fact, it was proposed that mitochondrial dysfunction interacts with all of the other hallmarks of aging described above (van der Rijt et al., 2020). It has long been known that mtDNA mutations accumulate with age (Kang et al., 2016; Li et al., 2019) and mitochondrial function declines with age, including altered mitochondrial respiration, reduced ATP, and altered metabolites. Importantly, mitochondria also produce reactive oxygen species (ROS) while generating ATP through oxidative phosphorylation (OXPHOS; Houtkooper et al., 2011; Lesnefsky & Hoppel, 2006). However, the excess production of ROS can induce oxidative damage to macromolecules due to the high chemical reactivity of ROS (Bratic & Larsson, 2013; López‐Otín et al., 2013). In particular, ROS can potentially damage both nuclear DNA as well as the small, specialized genome contained with the mitochondria itself, known as “mitochondrial DNA” (or mtDNA). This oxidation, in turn, negatively affects genomic integrity and can lead to chromosomal instability and, ultimately, cell death or disease. Telomeric DNA is especially vulnerable to oxidative damage and cannot be repaired easily in somatic cells; thus, oxidative stress tends to drive telomere attrition and to further accelerate cellular senescence (Fouquerel et al., 2019; Liu et al., 2020; Von Zglinicki, 2002).

In this study, we seek to better understand the mechanisms by which the accumulation of mtDNA mutations may contribute to cellular and oxidative damage that result in DNA instability and telomeric DNA damage, leading to premature aging. To answer this question, we have chosen a mutant mouse model (*Polg* ^*D257A/D257A*^, or “*Polg* ^*mut/mut*^”, hereafter referred to as “*Polg* ^*mut/mut*^”) that possesses a high mtDNA mutation rate and have been previously linked to both severe mitochondrial dysfunction as well as accelerated aging (Kujoth et al., 2005; Trifunovic et al., 2004). The results of these experiments strongly suggest that this accelerated mtDNA mutation rate causes increased 8‐oxoguanine (8‐oxoGua) levels, nuclear DNA strand breakage, and telomere shortening, and provide a plausible explanation for mitochondria‐related aging.

## 2 RESULTS

### 2.1 Pathogenic mutations in mtDNA accelerate DNA methylation aging in human

**FIGURE 1:**
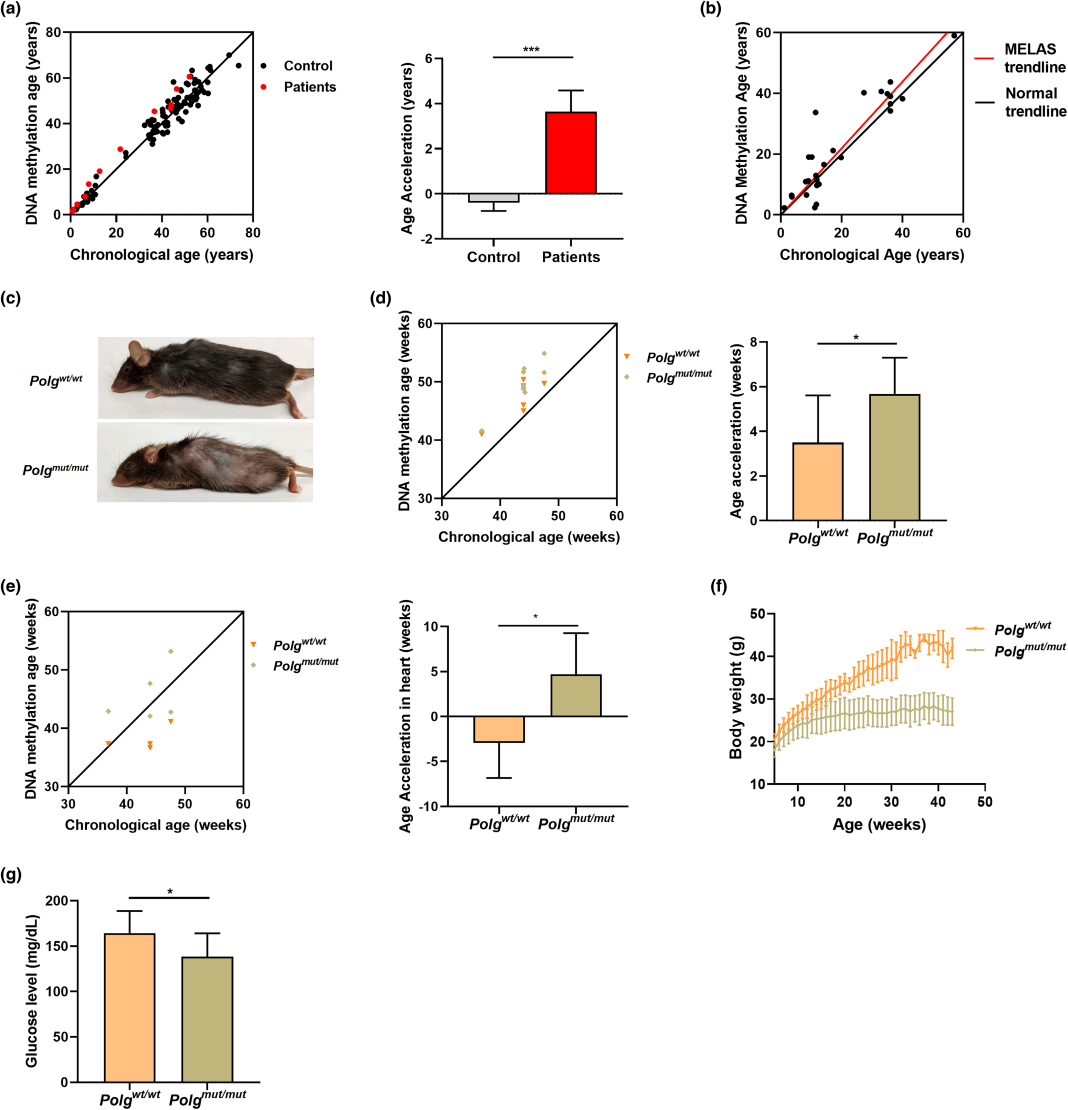
Pathogenic mitochondrial DNA mutations accelerated aging. The predicted DNA methylation age (DNAm) versus the chronological age with and the corresponding age acceleration (a) in 111 control samples (black) and 12 samples carrying known pathogenic mtDNA mutations (red, *n* = 12, including six patients carrying the m.3243A>G mutation, five carrying mtDNA mutations related to Leigh syndrome, and one carrying the m.8344A>G mutation related to myoclonic epilepsy with ragged red fibers (MERRF). (b) Additional DNA methylation analysis of samples from 30 patients carrying the pathological mtDNA mutation m.3243A>G. Note that the DNA methylation age of the MELAS patients (red line) is significantly accelerated relative to what would be expected if the DNA methylation age correlated precisely to chronological age (black line). The 95% confidence interval for the linear regression of the MELAS samples is indicated by the dotted lines. (*n* = 30). (c, f, g) *Polg* ^*mut/mut*^ mice display a distinct aging phenotype relative to *Polg* ^*wt/wt*^ siblings, including alopecia, kyphosis, and reduced subcutaneous fat (c), loss of body weight (f) (*n* ≥ 8), and lower glucose levels (g) (*n* ≥ 11). The predicted DNA methylation age versus the chronological age, and the corresponding age acceleration in blood samples (d) (*n* ≥ 6) and heart samples (e) (*n* ≥ 4). The data are presented as the mean ± S.D (mean ± SEM for panel a), with *p < 0.05, **p < 0.01, ***p < 0.001, and ****p < 0.0001

DNA methylation is an important epigenetic marker that varies with age and has received widespread attention in areas such as biological development, aging, and oncology. Horvath et al. have previously developed a DNA methylation‐based estimator of biological age (pan tissue clock) that applies to all human tissues (Horvath, 2013; Horvath et al., 2015, 2016; Horvath & Levine, 2015; Jones et al., 2015). The human pan tissue clocks (also referred to as “DNA Clocks”) have been developed to estimate biological age based on 353 methylation sites known as CpGs. Similar pan tissue clocks have also been developed for mice and other species (Horvath, 2013; Petkovich et al., 2017; Stubbs et al., 2017). Such DNA Clocks become accelerated in certain disease conditions as a reflection of the pathogenic damage of cells and tissues and premature aging. The relationship between the mitochondria and the DNA Clock is particularly intriguing, as mitochondrial dysfunction caused by mtDNA mutations has been previously shown to play an important role in global DNA methylation (Lopes, 2020). However, the specific relationship between the mitochondria and the DNA Clock remains an underexplored question. Therefore, to test the effect of mitochondrial dysfunction on DNA methylation, DNA samples from 123 patients were collected and analyzed for their epigenetic age based on DNA methylation clocks (Horvath, 2013; Horvath et al., 2016), including 12 samples carrying known pathogenic mtDNA mutations and 111 control samples lacking any known pathogenic mtDNA mutations. Six of the 12 patients with pathogenic mtDNA mutations carried the m.3243A>G mutation, five carried mtDNA mutations related to Leigh Syndrome, and one carried the m.8344A>G mutation related to myoclonic epilepsy with ragged red fibers (MERRF). The results showed that DNA methylation age in 12 patients with pathogenic mtDNA mutations was accelerated by more than 3 years relative to their expected age, while the control samples showed essentially no DNA methylation age acceleration (on average ~ 0.5 years less than their expected age; Figure 1a). These results were further confirmed in a follow‐up analysis of DNA methylation age in 30 additional patients carrying the pathogenic mtDNA mutation m.3243A>G that causes mitochondrial encephalopathy with lactic acidosis and stroke‐like episodes (MELAS). This single nucleotide mutation alters the mitochondrial tRNA^Leu(UUR)^ and reduces the activity of complexes I and IV, leading to a disruption in the translation of proteins encoded by the mitochondrial DNA (mtDNA). The resulting loss in oxidative phosphorylation activity leads to disease via decreased ATP production and increased ROS levels in the cell (Goto et al., 1992; Horváth et al., 2008; Kodaira et al., 2015; Li et al., 2019). The results from this cohort of 30 MELAS patients show that the m.3243A>G mutation accelerates DNA methylation age by an average of 2.6 years relative to chronological age (Figure 1b), in line with the results from the initial set of 12 patients with pathogenic mtDNA mutations. Together, these provide the first quantitative evidence that pathogenic mitochondrial DNA mutations can increase DNA methylation age in humans.

### 2.2 *Polg* mutation accelerates epigenetic and physical aging in mice

The *Polg* ^*mut/mut*^ mouse model carries an error‐prone allele of the gene encoding the mitochondrial DNA polymerase known as “polymerase gamma” (or “POLG” for short). This error‐prone version of POLG results in a higher rate of mutation in the mitochondrial DNA, which appears to be the major underlying cause of the accelerated aging phenotype that has been repeatedly shown in this model (Kujoth et al., 2005; Trifunovic et al., 2004). In addition, the *Polg* ^*mut/mut*^ mice show multiple distinct physical aging phenotypes, including alopecia, graying hair, kyphosis, reduced mobility, weight loss, and hypoglycemia (Figure 1c,f,g). Previous experiments in a separate “mtDNA depleter” model carrying an inducible, dominant‐negative mutation of POLG that interferes with mtDNA replication itself have also shown a similar premature aging phenotype—including hair loss and increased skin wrinkles—that can be reversed upon removal of the mutant protein (Singh et al., 2018). While the precise cellular and molecular underpinnings of this POLG‐related premature aging phenotype are not fully understood, the DNA Clock provides a means of quantifying that effect at the epigenetic level. To test whether the *Polg* ^*mut/mut*^ mutation also effects the epigenetic age of these mice, the DNA methylation age of blood samples from *Polg* ^*mut/mut*^ mice were analyzed using established methods in mice (Coninx et al., 2020; Stubbs et al., 2017). The results show that *Polg* ^*mut/mut*^ mice have a higher than expected DNA methylation age (2.18 weeks) and accelerated aging in blood samples as compared to *Polg* ^*wt/wt*^ mice (Figure 1d). Additional follow‐up analysis using a universal epigenetic clock that can estimate age across a variety of tissues and species also confirmed that *Polg* ^*mut/mut*^ mice shown a significant age acceleration (7.62 weeks) in heart tissue DNA relative to *Polg* ^*wt/wt*^ control mice (Figure 1e). This suggests that premature aging induced by *Polg* mutation is also detectable at the epigenetic and cellular level, in a manner consistent with the overt progeria phenotype observed in this mouse model. The results of the DNA methylation analysis indicate that the accumulation of mitochondrial mutations caused by *Polg* mutation indeed accelerated aging in the *Polg* ^*mut/mut*^ mice. This provides further corroboration for the epigenetic age acceleration observed in human patients with pathogenic mtDNA mutations.

### 2.3 *Polg* mutation increases 8‐oxoGua in urinary samples

**FIGURE 2:**
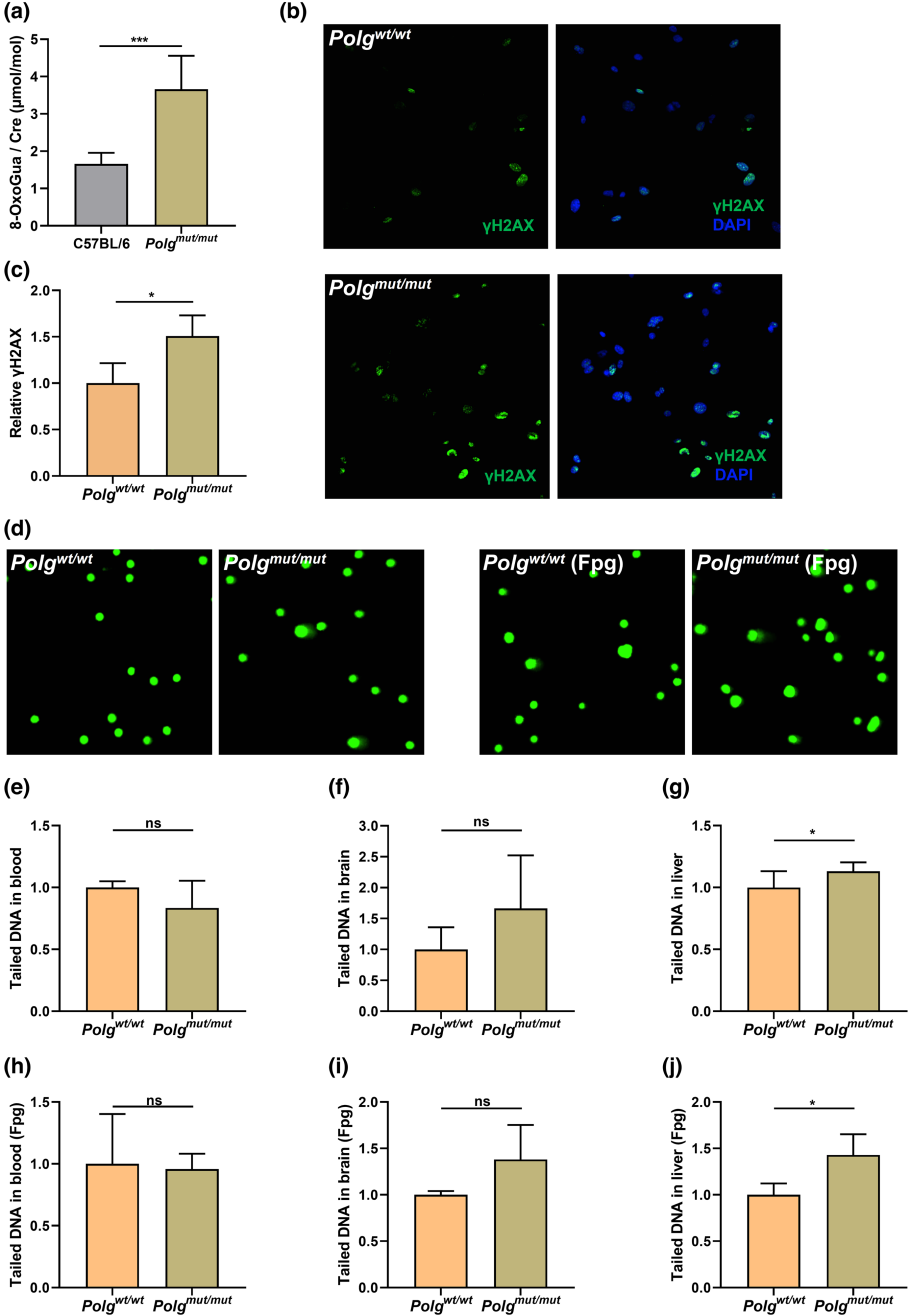
Elevated 8‐oxoguanine (8‐oxoGua) levels, DNA strand breakage, and oxidative DNA damage in *Polg* ^*mut/mut*^ mice. The 8‐oxoGua was quantified in urinary samples by ultraperformance liquid chromatography tandem mass spectrometry (UPLC‐MS/MS) (a) (*n* = 7). The γH2AX staining in *Polg* ^*wt/wt*^ (b up panel) and *Polg* ^*mut/mut*^ (b down panel) MEFs was assessed by immunofluorescence, and the number of γH2AX‐positive *Polg* ^*mut/mut*^ MEFs normalized with the number of γH2AX‐positive *Polg* ^*wt/wt*^ MEFs (c). Representative images of comet assay result with or without Formamidopyrimidine DNA glycosylase (Fpg) treatment in *Polg* ^*wt/wt*^ and *Polg* ^*mut/mut*^ mice (d). The levels of DNA strand breakage in blood (e), brain (f), and liver (g) cells were assessed by comet assay. The DNA strand breakage and oxidative DNA damage were also measured in blood (h), brain (i), and liver (j) by comet assay with Fpg treatment. Data are presented as the mean ± SD, with *p < 0.05, **p < 0.01, ***p < 0.001, ****p < 0.0001. (*n* ≥ 3)

The molecular mechanism of premature aging in the *Polg* ^*mut/mut*^ mutant mouse is not fully understood. Homozygous *Polg* ^*mut/mut*^ causes extensive mtDNA mutations, including random point mutations as well as linear and circular molecules with large deletions and multimers of the mtDNA control region in certain tissues (Hiona et al., 2010; Jang et al., 2018; Trifunovic et al., 2004; Vermulst et al., 2008; Williams et al., 2010). This high level of mtDNA mutations caused instability of the respiratory chain complexes and respiratory chain deficiency in *Polg* ^*mut/mut*^ mice (Edgar et al., 2009). Furthermore, accumulation of mitochondrial DNA mutations and deletions results in highly localized impairment of mitochondrial function and increased oxidative stress. This loss of energy‐producing function and the increase chemical damage resulting from oxidative stress are likely to be involved in the aging process with multiple targets (Fayet et al., 2002; Larsson, 2010; Lei et al., 2021; Liang et al., 2020). In particular, DNA molecules could be a major target of any increased oxidative stress originating from the mitochondria. Thus, in order to test this hypothesis, we first examined the level of the free nucleotide 8‐oxoGua, which is a well‐known and biologically relevant chemical species that results from the oxidation of free nucleotide guanine or guanine in DNA (Kanvah et al., 2010). To test whether *Polg* ^*mut/mut*^ mutation can result in increased oxidative damage to the free nucleotide pool, 8‐oxoGua levels were quantified in urinary samples by ultraperformance liquid chromatography tandem mass spectrometry (UPLC‐MS/MS) and the total 8‐oxoGua was normalized to the levels of creatinine in the same samples. The results show that the *Polg* ^*mut/mut*^ mutation significantly increases the level of 8‐oxoGua in urinary samples (Figure 2a). This result suggests that *Polg* ^*mut/mut*^ likely increases the level of total oxidative stress in the cell, and that this oxidative damage is further propagated to the pool of free nucleotides in the cell.

### 2.4 *Polg* mutation increases nDNA strand breakage and oxidative nDNA damage

Several methods have been reported to identify endogenous 8‐oxo‐2′‐deoxyguanosine (8‐oxodG) sites in the genome, such as OG‐Seq, Click‐code‐seq, AP‐seq, ChIP‐seq, TRAP‐seq, and OxiDIP‐seq (Amente et al., 2019; Ding et al., 2017; Fang & Zou, 2019; Hao et al., 2018; Poetsch et al., 2018; Wu et al., 2018). Using these methods, previous work has shown a strong correlation between 8‐oxodG sites (OxiDIP‐Seq) and γH2AX signals (ChIP‐Seq) of RefSeq genes, indicating that the oxidative DNA lesion potentially leads to a DNA damage response (DDR) activation that is typically associated with DNA double‐strand breaks (DSBs; Amente et al., 2019), strand breakage, telomere uncapping, and/or replication stress. Therefore, to determine whether the higher 8‐oxodG levels observed in the DNA molecules themselves are also associated with canonical DDR linked to DNA strand breakage, we have examined γH2AX levels and DNA damage in MEFs derived from *Polg* ^*mut/mut*^ by immunofluorescence. The results show that *Polg* ^*mut/mut*^ increases the number of γH2AX‐positive MEFs (Figure 2b,c), indicating that *Polg* ^*mut/mut*^ increases DDR and potentially DNA DSBs, and consistent with an increase of 8‐oxodG in nDNA.

DNA damage was further analyzed by combining comet assay with Formamidopyrimidine DNA glycosylase (Fpg) incubation in different cell types from *Polg* ^*mut/mut*^ mice. For these assays, cells are embedded in agarose before being lysed to release their DNA. Electrophoresis is then performed under alkaline conditions to convert AP sites and alkali‐labile DNA adducts into single‐strand breaks (SSBs), which are subsequently convert to DSBs under the alkaline conditions. Both SSBs and DSBs are then detected via the electrophoretic movement of the DNA fragments through the agarose. When the agarose is subsequently stained for DNA, undamaged DNA remains intact and runs as a coherent mass, while damaged DNA strand breaks migrate further than intact DNA, forming a “comet‐shaped” tail. Therefore, comet tails will tend to be observed in cells with DNA damage, with the percentage of DNA in the tails representing the degree of DNA damage. Using this assay, we show that the levels of DNA SSBs and/or DSBs are significantly higher in liver (Figure 2d–g), which suggests that *Polg* ^*mut/mut*^ are more likely to experience DNA strand breakage. To detect 8‐oxodG, we further treated the lysed cells with Fpg, an enzyme that converts 8‐oxodG and other oxidatively damaged residues into SSBs. The results show that the tailed DNA significantly increases after Fpg treatment in liver (Figure 2h–j), further suggesting that oxidatively damaged residues (including 8‐oxodG) are accumulating in *Polg* ^*mut/mut*^ DNA. Together, these results are consistent with our hypothesis that *Polg* ^*mut/mut*^ increases the 8‐oxoGua levels and oxidative damage to DNA.

### 2.5 *Polg* mutation decreases telomere length and increases telomere damage

Telomeres are the specialized DNA/protein complexes made of repetitive sequences (TTAGGG) that are present at the ends of chromosomes. Telomeres are bound by Shelterin proteins to protect telomeres (and, by extension, the rest of the chromosome) from damage and loss of critical DNA sequence information during mitosis (Martínez & Blasco, 2010). At the same time, the Shelterin proteins also prevent DNA DSBs repair proteins from falsely recognizing telomeres at chromosome ends as DNA breaks (De Lange, 2005). It is well‐documented that telomeres shorten with age due to the end replication problem; however, the molecular mechanisms that accelerate telomere shortening under oxidative stress conditions have yet to be fully elucidated. Since telomeres are GC rich, they are preferred targets of 8‐oxodG related DNA damage (Oikawa et al., 2001). This vulnerability to oxidative damage, combined with the observation that damaged telomere DNA appears to be repaired poorly by the cell, has been proposed to contribute to the telomere shortening observed in aging (Fumagalli et al., 2012; Hewitt et al., 2012; Von Zglinicki, 2002).

**FIGURE 3:**
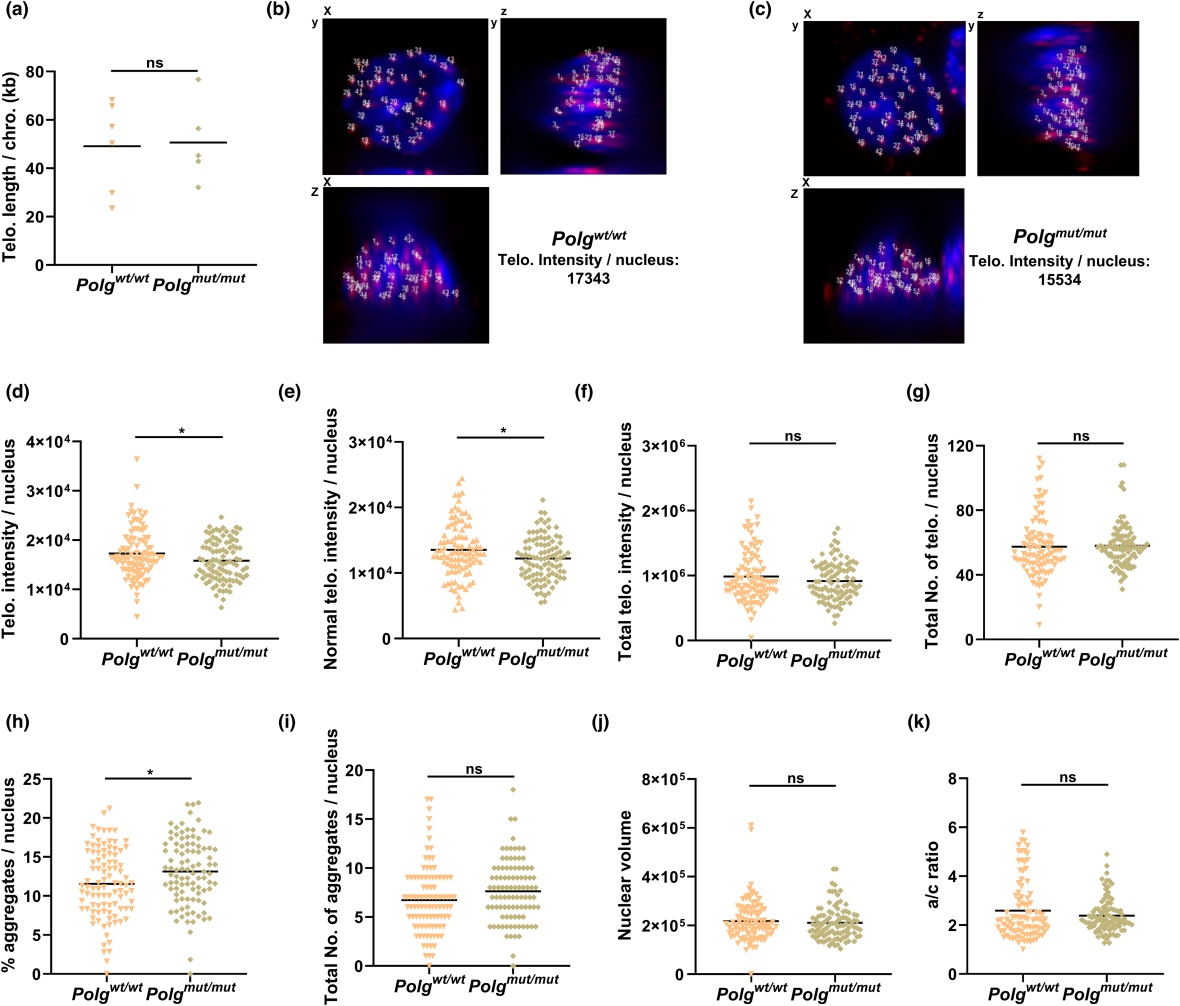
Shortened telomeres in *Polg* ^*mut/mut*^ mice. (a) The absolute telomere (Telo.) length in total blood DNA shows no overall difference when assessed by real‐time PCR. The data are presented as the means ± SD. *p < 0.05, **p < 0.01, ***p < 0.001. (*n* ≥ 5). (b–k) However, quantitative fluorescent in situ hybridization (Q‐FISH) results from lymphocytes of *Polg* ^*wt/wt*^ and *Polg* ^*mut/mut*^ mice indicate significant shortening of the telomeres in mutant lymphocytes. The representative average intensity of the Q‐FISH images is shown for a nucleus derived from a *Polg* ^*wt/wt*^ (b) and *Polg* ^*mut/mut*^ (c) cell. The blue signal represents DAPI staining, while the red signal represents staining for the telomere‐specific probe. (d–k) Quantitative measurements are shown for the average intensity of all telomeres signals (d), average intensity of normal telomeres signals (e), total intensity of telomere signals (f), total number of telomere signals (g), and percentage of telomere aggregates (h), total number of telomere aggregates (i). The average nuclear volume (j) and *a/c* ratio (K) are also shown as control. *p < 0.05, **p < 0.01, ***p < 0.001, ****p < 0.0001. (*n* ≥ 90)

**FIGURE 4:**
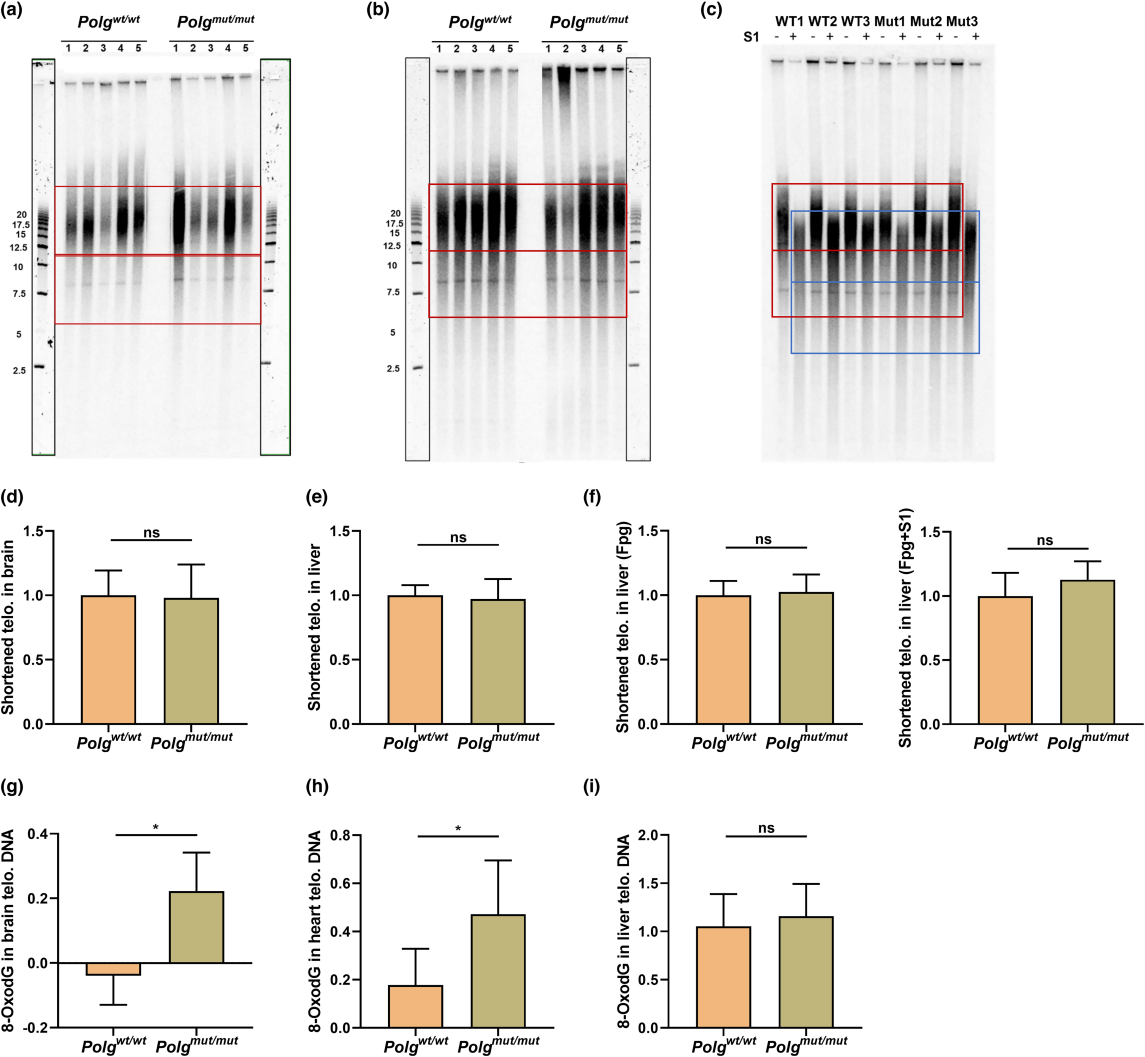
Assessment of oxidative DNA damage in telomeres. The Southern blot results of telomere length for brain and liver DNA samples are shown in (a) and (b), and quantifications of the shortened telomeric DNA are shown in (d) and (e). To quantify the shortened telomeric DNA, boxes were drawn in the lanes and the signal was quantified for the “bulk” of the telomeric DNA (the upper red boxes) and for the “tail” of the telomeric DNA (the lower red boxes) by Image J. A higher proportion of DNA in the “tail” fraction is indicative of degraded or shortened telomeric DNA. The Southern blot results of oxidative DNA damage in liver telomeric DNA are also shown following Fpg digestion with and without S1 treatment (c), and the quantifications of cleaved telomeric DNA for each treatment are shown in (f), with the red boxes representing the regions used to quantify Fpg treatment, and the blue boxes representing the regions used to quantify S1 treatment. The real‐time PCR results for brain, heart, and liver telomeric DNA with 8‐oxodG are also shown in (g), (h) and (i), respectively. The data are presented as the mean ± SD, with *p < 0.05, **p < 0.01, ***p < 0.001, ****p < 0.0001. (*n* ≥ 3)

Given the strong association with both aging and DNA damage and as a potentially sensitive target of 8‐oxodG DNA damage, telomeres may represent a major interest for mitochondria‐related aging. Furthermore, previous work shows telomeres are preferentially damaged following mitochondrial dysfunction caused by targeted singlet oxygen production in the mitochondria (Qian et al., 2019). To test whether the *Polg* ^*mut/mut*^ affects telomere length, real‐time PCR, Southern Blot, and Q‐FISH methods were used to detect changes in telomere length and integrity. First, the average telomere length was measured by real‐time PCR and Southern Blot. These initial results showed that there was no significant difference in telomere length (Figures 3a, 4a,b,d,e). However, this is not entirely unexpected considering the limitations to the sensitivity of real‐time PCR and the relatively long telomeres present in mice, which are 5–10 times longer than those in humans (Calado & Dumitriu, 2013). Thus, quantitative fluorescent in situ hybridization (Q‐FISH) with high sensitivity was used to further measure the telomere length and damage from individual nuclei. In this approach, the telomeric signal intensity represents the length of the telomeres in arbitrary units, as the intensity of the peptide nucleic acid (PNA) probe hybridization signal is directly proportional to the length of the telomeric DNA (Poon et al., 1999). Thus, the average intensity of all of the telomeric objects detected within a given nucleus represents the average length of the telomeres within that nucleus. Telomere aggregates are another important feature of an aberrant telomere profile. When the telomeres get shorter and shorter, the physiological binding of the shelterin proteins is compromised, and the telomeres are left uncapped. Uncapped telomeres have the tendency to fuse together, forming telomere aggregates (TAs). In a normal cell, one would expect to observe approximately 92 telomere signals in interphase phase (in other words, two per chromosome). Any number significantly different from this may be indicative of aneuploidy (gain or loss of chromsomes) and/or the presence of interstitial telomeric signals. The number of TAs per nucleus can be used to measure the number of telomere clusters. An increase in the number of telomere clusters indicates that telomeres are close together, partly caused by increased telomere fusion or potentially homologous recombination, which is also associated with increased chromosome instability (Adam et al., 2019; Louis et al., 2005).

The results of our Q‐FISH analysis demonstrate that *Polg* ^*mut/mut*^ mutant cells show a decrease in their average telomere intensity (Figure 3b–f). Specifically, the average intensity of total telomere signals (Figure 3d), average intensity of normal telomere signals (Figure 3e), and total intensity of telomere signals (Figure 3f) were quantified (the normal telomere signals were obtained by removing abnormally long and short telomeres from total telomeres). The results indicate that the telomere length is significantly shorter in *Polg* ^*mut/mut*^ mutant mice. The total number and the percentage of aggregates were also increased in *Polg* ^*mut/mut*^ cells (Figure 3h,i), indicating that *Polg* ^*mut/mut*^ mutation increases telomere clusters and potentially telomere fusions. On the contrary, the total number of telomeres (Figure 3g) appears to be unchanged relative to wildtype controls, indicating that there is no complete telomere loss or polyploidy. The *a/c* ratio defines the nuclear space occupied by telomeres—as represented the ratio between the a‐axes and c‐axes (the *a/c* ratio)—which vary based on the different stages of the cell cycle (G0/G1, S, G2, etc.). In our results, the *a/c* ratio (Figure 3k) shows no difference in *Polg* ^*mut/mut*^ cells, confirming that the cells are at the same cell cycle stage (Gadji et al., 2010; Mai, 2010; Mai & Garini, 2006; Mathur et al., 2014; Vermolen et al., 2005). Furthermore, since average nuclear volume (Figure 3j) is also unchanged relative to controls, telomere intensity (Figure 3d–f) is unlikely to be due to other cellular process. Together, these results show that the average telomere lengths decrease, and that the number of telomere clusters (a proxy for telomere instability) increases in *Polg* ^*mut/mut*^ mutant cells. This suggests that the observed increase in 8‐oxodG lesions in *Polg* ^*mut/mut*^ is associated with increased telomere instability, resulting in telomere shortening, promoting telomere damage, and possibly further compromising the stability of the genome.

### 2.6 Telomere shortening is correlated with 8‐oxodG mediated DNA oxidation and breakage

To further test whether telomeric DNA shortening (Figure 3d,e) and clustering (Figure 3h,i) is related to increased 8‐oxodG levels, telomeric DNA was further analyzed by Southern blot and real‐time PCR combined with Fpg and S1 enzymes. If the telomere restriction fragments contain an 8‐oxodG lesion, then Fpg will convert 8‐oxodG to an SSB, which is then converted to a DSB by S1 nuclease. This causes the cleaved telomere to migrate faster. The results of Southern blot did not show any major differences between *Polg* ^*mut/mut*^ and *Polg* ^*wt/wt*^, and *Polg* ^*mut/mut*^ in the fraction of cleaved telomeric DNA from the liver (Figure 4c,f). However, the results of real‐time PCR analysis show that the levels of 8‐oxodG in brain and heart telomeric DNA increase significantly after Fpg incubation in *Polg* ^*mut/mut*^ mice (Figure 4g,h), although there was again no significant difference in liver DNA samples (Figure 4i). These results suggest that 8‐oxodG is modestly increased alongside the previously observed telomere shortening in *Polg* ^*mut/mut*^ mice, with the effect more or less pronounced depending on the tissue.

### 2.7 *Polg* mutation increases mtDNA strand breakage and oxidative damage

The results described above show that *Polg* ^*mut/mut*^ increases 8‐oxodG levels and likely affects the integrity of nuclear DNA including telomeres. Given its physical proximity to the mitochondria, mtDNA might be expected to be even more susceptible to oxidative damage relative to nDNA, since the pool of nucleotides used to replicate mtDNA and the existing mtDNA molecules would likely have a higher percentage of their guanines converted into 8‐oxodG. If so, one would expect a higher level of 8‐oxodG in the mtDNA and a corresponding increase in mtDNA strand breakage.

**FIGURE 5:**
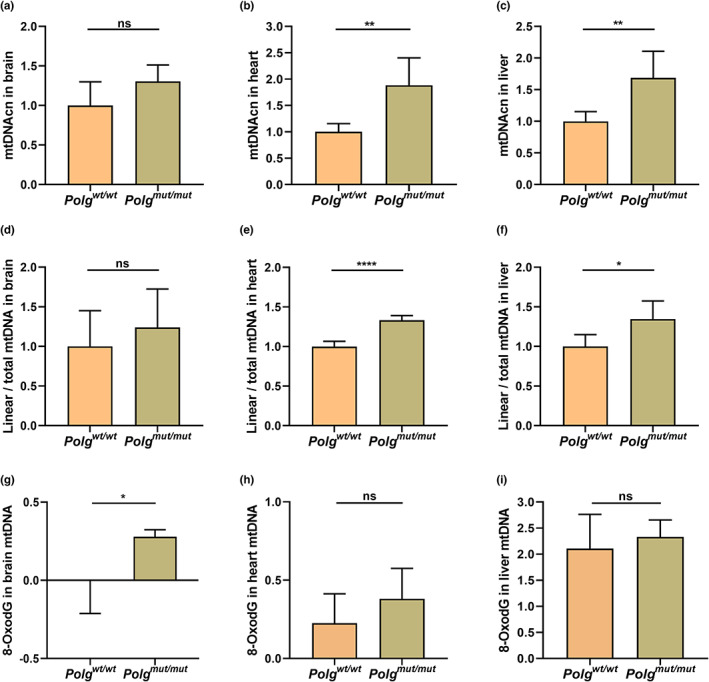
Mitochondrial DNA copy number (mtDNAcn) and oxidative DNA damage in mtDNA. The total mtDNAcn in brain (a), heart (b), and liver (c) was determined by real‐time PCR. The linear mtDNAcn in brain (d), heart (e), and liver (f) was also determined by real‐time PCR following exonuclease V incubation. The 8‐oxodG levels in brain (g), heart (h), and liver mtDNA (i) were determined by real‐time PCR following Fpg incubation. The data are presented as the mean ± SD, with *p < 0.05, **p < 0.01, ***p < 0.001, ****p < 0.0001. (*n* ≥ 3)

To test the hypothesis that *Polg* ^*mut/mut*^ increases mtDNA strand breakage, we used real‐time PCR to detect mtDNA copy number (mtDNAcn) with or without exonuclease V treatment in different tissues. Exonuclease V is a DNA exonuclease which specifically degrades linear DNA molecules while leaving circular DNA molecules intact. Thus, by clearing the nuclear DNA and linear mtDNA from each sample via exonuclease V incubation, only circular molecules left (such as intact mtDNA) in the post‐treatment sample. In this way, the relative content of linear mtDNAcn before and after exonuclease V treatment can be calculated by comparing cycle threshold (Ct) values, which represent the number of amplification cycles required for the fluorescent signal to cross the threshold separating background fluorescence from a true signal in real‐time PCR. In general, a smaller Ct value indicates a larger relative amount of target DNA present in the sample, while larger Ct values indicate that less of the target DNA is present in the sample. Our results show that *Polg* ^*mut/mut*^ increases both the total mtDNAcn (Figure 5b,c) and the linear mtDNAcn (Figure 5e,f **)**. There were similar trends in the brain, but not at a statistically significant level (Figure 5a,d). The ratio of the linear mtDNA to total mtDNA also increased, suggesting that the physical integrity of the mtDNA has been compromised. It was also found that linear mtDNAcn increased in *Polg* mutator mice by 2D‐IMAGE (two‐dimensional intact mtDNA agarose gel electrophoresis; Kolesar et al., 2014). The increase in the total mtDNAcn may suggest the cells attempt to compensate for the increasingly damaged, fragmented, and functionally compromised mtDNA molecules (Giordano et al., 2014; Rausser et al., 2021; Yu‐Wai‐Man et al., 2010). To verify whether the increase of linear mtDNAcn in *Polg* ^*mut/mut*^ is caused by the higher levels of oxidative damage, the 8‐oxodG levels were measured in mtDNA by real‐time PCR combined with Fpg incubation. The results show that 8‐oxodG increases significantly in brain mtDNA (Figure 5g). However, while the results showed an overall trend that *Polg* ^*mut/mut*^ increases the 8‐oxodG levels in mtDNA of heart and liver, the difference was not statistically significant (Figure 5h,i). These results suggest that 8‐oxodG in *Polg* ^*mut/mut*^ increases mtDNA strand breakage via increased oxidative damage variable in a tissue‐specific manner and in a tissue‐specific response.

## 3 DISCUSSION

**FIGURE 6:**
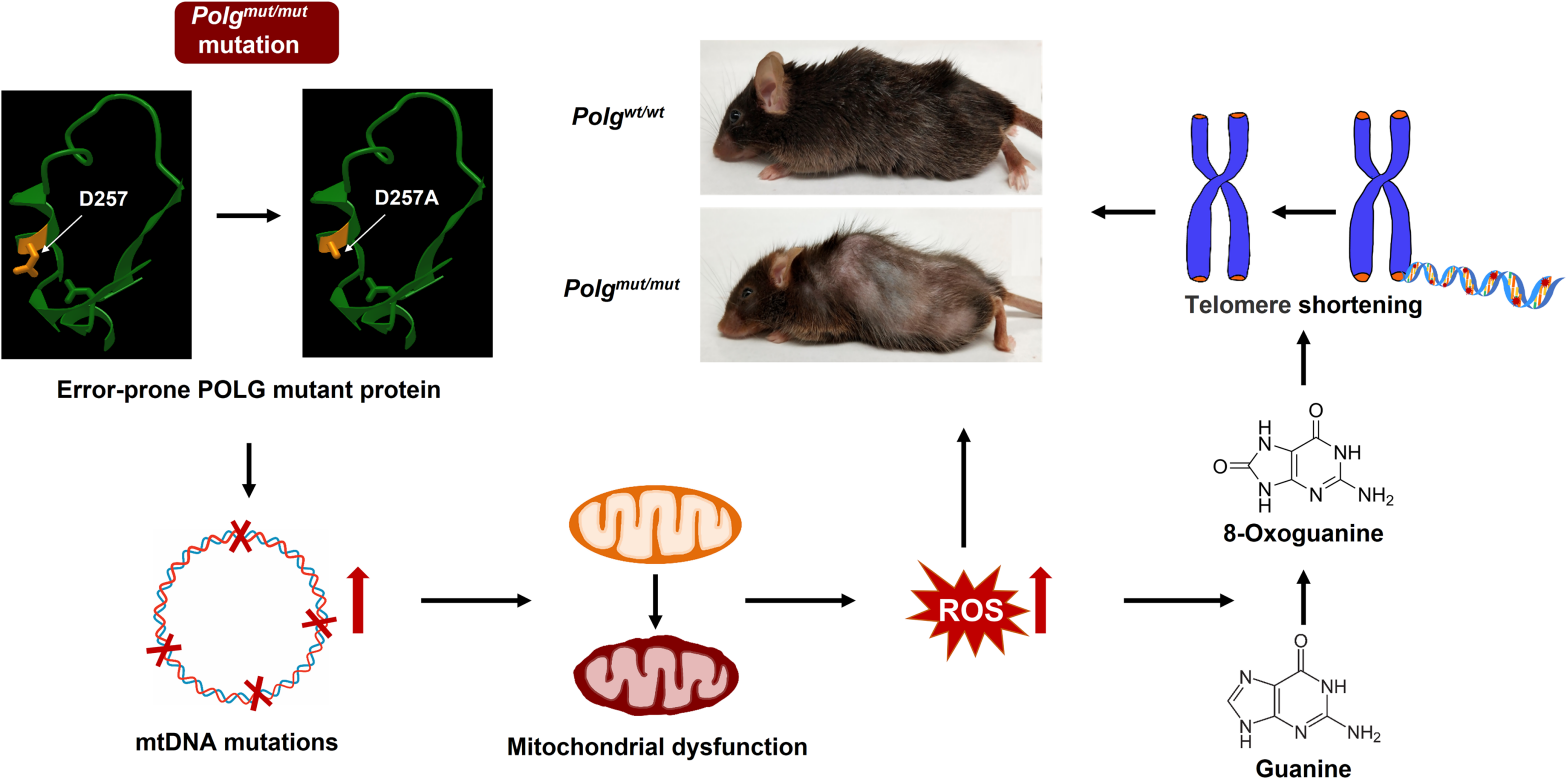
Model of *Polg* ^*mut/mut*^ mutation leading to increased ROS, higher 8‐oxoGua levels, and telomere shortening. The protein structure for the conserved exonuclease domain II of POLG (sites 265–297) was cited from iCn3D, excluding the D257A mutation (Wang et al., 2020)

It has been proposed that oxidative damage to various biomolecules (protein, lipids, nucleic acid, etc.) is a major cause of the progeria phenotype observed in *Polg* ^*mut/mut*^ mice. However, the question of whether ROS levels are truly increased in *Polg* ^*mut/mut*^ mice—as well as the role of any putative increase in ROS to their aging phenotype—remains controversial in the existing literature (Hiona et al., 2010; Kujoth et al., 2005; Kukat et al., 2014; Lei et al., 2021; Logan et al., 2014; Maclaine et al., 2021; Trifunovic et al., 2005; Zsurka et al., 2018). Interestingly, despite the extensive investigation of various oxidized biomolecules in the *Polg* ^*mut/mut*^ mouse, one class of molecules that has received less attention to date is oxidized nucleotides. For this reason, we have focused on this question of the role of nucleotide oxidation in the *Polg* ^*mut/mut*^ mouse, based on the existing literature suggesting that guanine nucleotides would be a particularly vulnerable targets for oxidative stress that results from the *Polg* mutation. In turn, the increased 8‐oxoGua produced by this oxidative damage have a particular effect on the stability of DNA, which causes telomere shortening, nDNA damage, and mtDNA integrity. Together, all of this would fit the hypothesis that mitochondria, and particularly oxidative stress, play a central role in the aging process. Telomere damage could be secondary to mitochondrial dysfunction due to the increased production and incorporation of 8‐oxoGua into the DNA (Figure 6). Since the increased 8‐oxoGua can also compromise mtDNA integrity and further damage the mitochondrial function, the most likely result is the production of even more ROS and an increase in 8‐oxoGua. This is a likely to be vicious cycle and breaking this vicious cycle may be a potential target for mitigating aging. In that light, there are several specific aspects of these results that bear further reflection and closer examination in the future.

First, the DNA methylation age analysis described here provides the first quantitative evidence that single nucleotide, pathogenic changes in mtDNA are sufficient to accelerate cellular aging in humans. In addition, the results from blood samples from *Polg* ^*mut/mut*^ show a similar pattern of DNA methylation age acceleration, providing a strong confirmation of the link between mitochondrial pathogenesis and aging. The fact that the premature aging caused by mtDNA mutations can be observed so dramatically in blood samples is also consistent with previous work on the *Polg* ^*mut/mut*^ model showing that *Polg* mutation triggers inflammation and alterations in circulating leukocyte populations (Lei et al., 2021; West, 2017) as well as anemia due to the loss of erythrocytes (Ahlqvist et al., 2015; Chen et al., 2009). Given these results, it will be interesting to see whether similar patterns of inflammation and altered cell populations are recapitulated in other models of mitochondrial disease such as MELAS alongside the shared epigenetic age acceleration shown here, and whether this might be an underappreciated mechanism of shared pathogenesis for these diseases. The question of the direct relationship between 8‐oxoGua and DNA methylation also bears further investigation, as it has been previously shown that DNA methylation is negatively correlated with the levels of 8‐oxoGua in DNA, likely because the 8‐oxoGua residue interferes with the methylation of its adjacent cytosine reside (Guz et al., 2008). In addition, increased ROS has been shown to cause changes in global methylation that can silence DNA damage repair genes, including the 8‐oxoGua base excison repair enzyme OGG (Haldar et al., 2016), providing a reciprocal mechanism by which DNA methylation may also influence 8‐oxoGua levels in DNA.

Second, this paper has shown the unique importance of the oxidation of guanine nucleotide as a readout for mitochondrial dysfunction. The guanine residue has the lowest oxidation potential among the DNA bases, and 8‐oxoGua is one of the major and abundant oxidative DNA bases resulting from ROS modification (Steenken & Jovanovic, 1997). 8‐OxoGua can be recognized and repaired by 8‐oxoguanine DNA glycosylase (hOGG1), which can remove 8‐oxoGua and cleave the DNA backbone (Bruner et al., 2000). In general, the unrepaired 8‐oxoGua will mismatch with adenine residues during DNA replication, resulting in G:C to T:A transversion (Steenken & Jovanovic, 1997); however, there is evidence that POLG is less susceptible to this kind of misincorporation than other DNA polymerases (Graziewicz et al., 2007). The level of 8‐oxodG is an established biomarker of oxidative stress, and the accumulation of 8‐oxodG is associated with inflammation, cardiovascular, cancer, and aging (Cooke & Evans, 2007; Cooke et al., 2003; Li et al., 2016). Our results show that *Polg* ^*mut/mut*^ increases the total 8‐oxoGua in urinary samples and the 8‐oxodG in DNA. The fact that increased 8‐oxodG damages the DNA is further corroborated by the results of the γH2AX staining and comet assay showing increased DNA damage and fragmentation in *Polg* ^*mut/mut*^ mice. This supports the long‐running hypothesis that premature aging is caused (at least in part) by increasing chronic oxidative stress.

Finally, these results establish a previously unexplored relationship between the progeria phenotype of the *Polg* ^*mut/mut*^ mouse and telomere damage. The length of telomeres has, of course, been previously shown to decrease with age in humans (Blasco, 2007). Telomeres appear to be preferred targets of DNA damage, and the protein complex known as Shelterin may impair the repair of damaged DNA in some contexts, resulting in persistent DDR in telomeric DNA (Fumagalli et al., 2012; Hewitt et al., 2012; Palm & de Lange, 2008). In addition, Liu et al. showed that higher levels of ROS lead to telomere attrition and loss, chromosome fusion and breakage (Liu et al., 2002). Fouquerel et al. have also developed a chemoptogenetic tool to selectively produce 8‐oxoGua on telomeric DNA. The results of this analysis showed that the chronic induction of telomeric 8‐oxoGua shortens telomeres, which ultimately drives telomere crisis (Fouquerel et al., 2019). The accumulation of oxidative damage in telomeric DNA (whether by ROS produced by dysfunctional mitochondria or by other sources) thus causes telomere instability over time (Barnes et al., 2019; Qian et al., 2019). Our results support this conclusion, as *Polg* ^*mut/mut*^ mice show increased 8‐oxoGua and significantly shortened telomeres. While the differences in telomere length as measured by real‐time PCR and Southern blot were not sufficient to reach statistical significance, the results for the much more sensitive Q‐FISH analysis (while still relatively modest in magnitude) indicate that telomeres are significantly compromised over time, probably associated with the increased 8‐oxoGua. Compared with siblings, *Polg* ^*mut/mut*^ decreases the average intensity of telomeres signals by ~8.44% and increases the percentage of telomere aggregates by ~13.56% in mice 36 weeks of age or older. Both results indicate that *Polg* ^*mut/mut*^ accelerates telomere shortening and promotes telomere damage, making telomere DNA unstable and thereby shortening telomere length. Of course, the relatively small shifts in telomere length observed in these experiments provide one major caveat in the interpretation of these results, particularly given the relatively large size of mouse telomeres relative to humans (Kipling & Cooke, 1990). The modest decreases observed may also be suggestive of a gradual, but continual accumulation of DNA damage and telomere shortening, which is consistent with the slow but inexorable process of aging as opposed to a more rapid or sudden pathogenic condition. More importantly, it is likely that the effects of increased 8‐oxodG are not just confined to the telomeres, but contribute to genomic instability outside of the telomeric regions as well. In this sense, the shortening of the telomeres observed in the *Polg* ^*mut/mut*^ mice may just be an indicator of a more important underlying phenomenon of global DNA damage and fragility that may help drive the aging phenotype in these mice, consistent with our results.

In conclusion, these results provide multiple lines of evidence that mtDNA mutation associated with the accumulation of chronic oxidative stress is the center of aging by increasing nDNA and mtDNA strand breakage, and telomere shortening. Our results provide a plausible link between mitochondrial dysfunction and previously identified hallmarks in aging such as DNA methylation, telomere shortening, and oxidative stress. These are particularly important and timely insights given the aging of the world population that is expected to occur in the coming decade, and may help guide future focus to unravel the mitochondrial function and aging.

## 4 EXPERIMENTAL PROCEDURES

### 4.1 Experimental animals

*Polg* ^*wt/mut*^ mice were purchased from Jackson Laboratories (Jackson Lab Strain #017341) and maintained on a C57BL/6J background. The mice were maintained by Cincinnati Children's Hospital Medical Center (CCHMC) Veterinary Services and the UB's Division of Comparative Medicine and Laboratory Animal Facilities. Mice were housed in a temperature‐controlled room (24 ± 2°C) with a 12‐h light/12‐h darkness cycle. All procedures were approved by the Children's Hospital Research Foundation Institutional Animal Care and Use Committee, and the UB's Division of Comparative Medicine and Laboratory Animal Facilities and Use Committee.

### 4.2 DNA methylation age analysis

For analysis of DNA methylation age in human, patients were recruited, and consent was obtained according to the protocol approved by the Institutional Review Board of CCHMC (approval Study ID: 2013‐7868). Full details of the DNA methylation analysis for both mouse and patient DNA samples can be found in the Supporting Information attachment.

### 4.3 Glucose level

Glucose levels were detected by Accu‐Chek Guide Me glucose meter and test strips (Roche Diabetes Care). Blood for glucose measurements was drawn from the tail vein of mice.

### 4.4 8‐OxoGua quantification in urinary samples by UPLC‐MS/MS

Mouse urinary samples were collected in microcentrifuge tubes using the manual bladder palpation method (Nie et al., 2013) and stored at −80°C until they were analyzed. Full details of the UPLC‐MS/MS analysis of 8‐OxoGua levels in urinary samples can be found in the Supporting Information attachment.

### 4.5 Comet assay

The DNA damage and oxidation in single cells were detected by comet assay. The protocol was performed using the OxiSelect™ Comet Assay Kit (Cell Biolabs, Inc., Cat # STA‐351), according to the manufacturer's instructions. Additional experimental details can be found in the Supporting Information attachment.

### 4.6 γH2AX assay

The γH2AX was detected in Mouse Embryo Fibroblasts (MEFs). MEFs were harvested from embryos from *Polg* ^*wt/mut*^ female mice 14–15 days after the appearance of the copulation plug, as previously described (Durkin et al., 2013). MEFs were cultured in high glucose DMEM (Gibco) with 10% fetal bovine serum (Gibco) and 1% Antibiotic‐Antimycotic (Invitrogen). All cell lines were maintained at 37°C in a 5% CO_2_ incubator. Additional details regarding the immunofluorescence imaging of MEFs can be found in the Supporting Information attachment.

### 4.7 Quantification of telomere length by real‐time PCR

Genomic DNA of blood samples was extracted with the DNeasy Blood & Tissue Kit (Qiagen, Cat # 69504). The absolute telomere length was detected by real‐time PCR quantification assay kit (ScienCell, Cat # M8918), according to the manufacturer's instructions.

### 4.8 Quantification of telomere length by southern blot

Genomic DNA was extracted from tissues using the QIAGEN Tip‐100 Kit (Qiagen, Cat # 10223) according to the manufacturer's instructions. Two antioxidant chemicals, butylated hydroxytoluene (Sigma, Product # PHR1117; DMSO solvent) and deferoxamine mesylate (Sigma, Product # D9533; Water solvent), were added to the G2 lysis buffer at a final concentration of 100 mM each to prevent accidental oxidation of the DNA during the DNA extraction process. The detection of telomere length and 8‐oxoGua lesions in telomeric DNA were performed according to the previously described method (Fouquerel et al., 2019). To quantify the shortened telomeric DNA, boxes were drawn in each set of lanes to define the “bulk” and “tail” fractions (representing intact versus degraded telomeric DNA, respectively), and the signal for each fraction was quantified by Image J.

### 4.9 Quantification of telomere length by Q‐FISH

Telomere length was measured using splenic lymphocytes. For the spleen preparation, the spleen was minced, and the lymphocyte cells were washed out of the spleen tissue using with RPMI1640 medium. The lymphocytes were then resuspended in RPMI1640 medium, placed in blood collection tubes, and mixed with TransFix stabilization solution (Cytomark, Cat # TFB‐01‐10) at a 1:5 ratio to prevent coagulation. The tubes were gently inverted ten times to ensure a thorough mixture and then shipped to the cytology laboratory for processing. Cells were then prepared for Q‐FISH analysis, image acquisition, and TeloView software (Vermolen et al., 2005; Telo Genomics, Toronto, ON, Canada) as described in the Supporting Information attachment.

### 4.10 Quantification of total mtDNAcn and linear mtDNAcn by real‐time PCR

Total DNA was extracted using the DNeasy Blood & Tissue Kit (Qiagen, Cat # 69504) according to the manufacturer's instructions. Full details of the quantification of total mtDNAcn and linear mtDNAcn by real‐time PCR can be found in the Supporting Information attachment.

### 4.11 Quantification of 8‐oxodG levels in mtDNA and telomeric DNA by real‐time PCR

Full details of the quantification of 8‐oxodG levels in mtDNA and telomeric DNA by real‐time PCR can be found in the Supporting Information attachment.

## AUTHOR CONTRIBUTIONS

T.Y. and J.S. were involved in the conception, design, data generation, data acquisition, data interpretation, and manuscript writing. W.L. was involved in the design, data acquisition, data interpretation, and data analysis. R.B and P.L.O. performed telomere detection by Southern Blot. L.W. and S.M. performed telomere detection by Q‐FISH. S.H. performed the DNA methylation age analysis for the human samples. T.H. was involved in the conception, design, data interpretation, and manuscript writing.

## CONFLICT OF INTEREST

S.M. co‐founded Telo. Genomics Corp, is a shareholder, Director, and Chair of the Clinical and Scientific Advisory Board of the Company. The other authors declare no conflict of interest.

## Supporting information


Appendix S1

## Data Availability

The data that support the findings of this study are available on request from the corresponding author. The patient data are not publicly available due to privacy or ethical restrictions.
