# Supplementary material for: Premature aging is associated with higher levels of 8‐oxoguanine and increased DNA damage in the Polg mutator mouse
Source: Aging Cell. 2022 Aug 22;21(9):e13669. doi: 10.1111/acel.13669 (PMC9470903; doi:10.1111/acel.13669)
Supplement: Supplementary file 1 — Appendix S1 [file ACEL-21-e13669-s001.docx]

**Supplemental Material**

**Premature aging is associated with higher levels of 8-oxoguanine and increased DNA damage in the Polg mutator mouse**

Tenghui Yu^1,2,3,†^ , Jesse Slone^1,3,†^, Wensheng Liu^1^, Ryan Barnes^4^, Patricia L. Opresko^4^, Landon Wark^5^, Sabine Mai^5^, Steve Horvath^6^, Taosheng Huang^1,3,^*

1 Department of Pediatrics, University at Buffalo, 1001 Main Street, Buffalo, NY 14203, USA.

2 Human Aging Research Institute, School of Life Science, Nanchang University, Nanchang 330031, Jiangxi Province, China.

3 Division of Human Genetics, Cincinnati Children's Hospital Medical Center, Cincinnati, OH 45229, USA.

4 Department of Environmental and Occupational Health, University of Pittsburgh Graduate School of Public Health, and UPMC Hillman Cancer Center, Pittsburgh, PA 15232, USA.

5 CancerCare Manitoba Research Institute, The Genomic Center for Cancer Research & Diagnosis, University of Manitoba, Winnipeg, Manitoba R3E 0V9, Canada.

6 Human Genetics, David Geffen School of Medicine, University of California Los Angeles, Los Angeles, CA 90095, USA.

* Corresponding author. Email: thuang29@buffalo.edu (T.H.)

† These authors contributed equally to this work.

**Detailed Experimental Procedures**

**Experimental Animals**

Polg^wt/mut^ mice were purchased from Jackson Laboratories (Jackson Lab Strain #017341) and maintained on a C57BL/6J background. The mice were maintained by Cincinnati Children’s Hospital Medical Center (CCHMC) Veterinary Services and the UB’s Division of Comparative Medicine and Laboratory Animal Facilities. Mice were housed in a temperature-controlled room (24 ± 2°C) with a 12-hour light/12-hour darkness cycle. All procedures were approved by the Children’s Hospital Research Foundation Institutional Animal Care and Use Committee, and the UB’s Division of Comparative Medicine and Laboratory Animal Facilities and Use Committee.

**DNA methylation age analysis**

For analysis of DNA methylation age in human, patients were recruited, and consent was obtained according to the protocol approved by the Institutional Review Board of CCHMC (approval Study ID: 2013–7868). To calculate predicted DNA methylation age, blood DNA samples were obtained from 12 patients with verified pathogenic mtDNA mutations and 111 “control” individuals without pathogenic mitochondrial mutations. The DNA samples were shipped to the UCLA Neuroscience Genomics Core (UNGC) to further analyze DNA methylation age. The predicted DNA methylation age was then compared with the known chronological age for each sample to calculate the relative “age acceleration” for each sample. The calculation of DNA methylation age was performed according to the previously published method (Horvath, 2013; Horvath et al., 2016; Horvath & Levine, 2015). For the follow-up DNA methylation analysis of the 30 additional patients carrying the pathological mtDNA mutation m.3243A>G, blood DNA was assessed for DNA methylation age according to Zymo Research’s proprietary DNAge® predictor for humans.

For analysis of DNA methylation age in mouse blood, blood samples were collected from Polg^wt/wt^ and Polg^mut/mut^ mice at 36-47 weeks. Blood samples were stored in the EDTA tubes before extraction. Genomic DNA was extracted with the DNeasy Blood & Tissue Kit (Qiagen, Cat # 69504). The genomic DNA extracted from blood samples were shipped to Zymo Research (Irvine, CA) to further analyze DNA methylation age. Bisulfite conversion was performed using the EZ DNA Methylation-Lightning™ Kit (Cat. No. D5030) according to the standard protocol. Samples were then enriched for sequencing of >2,000 age-associated CpG loci, which identified using genome-wide reduced representation bisulfite sequencing (RRBS). DNA methylation values of blood samples were obtained from the sequence data and used to assess DNA age according to Zymo Research’s proprietary DNAge® predictor for mice (Coninx et al., 2020). Finally, for the array-based analysis of DNA methylation data from mouse heart tissue, the DNA samples were shipped to the UCLA Neuroscience Genomics Core (UNGC) to further analyze DNA methylation age using the mammalian methylation array (HorvathMammalMethylChip40), which provides deep coverage of up to 36K highly conserved CpGs in mammals (Arneson et al., 2021). The chip manifest file can be found at Gene Expression Omnibus (GEO) at NCBI as platform GPL28271. The SeSaMe normalization method was used to define beta values for each probe (Zhou, Triche Jr, Laird, & Shen, 2018). For estimation of DNA methylation age in the heart tissues, we used the pan tissue clock for mice, as described previously (Mozhui et al., 2021).

**Glucose level**

Glucose levels were detected by Accu-Chek Guide Me glucose meter and test strips (Roche Diabetes Care). Blood for glucose measurements was drawn from the tail vein of mice.

**8-OxoGua quantification in urinary samples by UPLC-MS/MS**

Mouse urinary samples were collected in microcentrifuge tubes using the manual bladder palpation method (Nie et al., 2013) and stored at -80°C until they were analyzed. The levels of 8-oxoGua and creatinine (Cre) in urinary samples were quantified using an Agilent 1290 UHPLC coupled to an Agilent 6495 QQQ MS instrument operated in the mode of multiple-reaction monitoring (MRM)/MS with (+) ion detection. A mixed standard solution of 8-oxoGua and creatinine was prepared with an internal standard (IS) solution of 8-oxoGua-^13^C3 and Cre-D3. This solution was serially diluted to make calibration solutions in a range of 0.0002 to 20 nmol/ml.

To perform the UPLC-MS/MS analysis, urinary samples were first thawed and vortexed. 20 µL of each sample was then diluted 500 times with the IS solution for quantitation of 8-oxoGua. The sample solution was further diluted 250 times with the IS solution for quantitation of creatine. After sonication and centrifugation, 10 µL aliquots of the supernatant of each sample and the calibration solutions were injected to run UPLC-MRM/MS on a HILIC column (2.1 x 100 mm, 1.7 µm) with an ammonium formate buffer and acetonitrile as the mobile phase for binary gradient elution (efficient gradient 90% acetonitrile to 10% in 10 min) at 0.35 ml/min and 30°C.

Concentrations of 8-oxoGua and creatine were calculated from the constructed linear-regression curve of each compound with internal standard calibration and using the analyte-to-IS peak ratios measured from urinary sample solutions.

**Comet assay**

The DNA damage and oxidation in single cells were detected by comet assay. The protocol was performed using the OxiSelect^TM^ Comet Assay Kit (Cell Biolabs, Inc, Cat # STA-351), according to the manufacturer’s instructions. Briefly, cells were harvested by mincing a small piece of tissue in 1 ml of ice-cold PBS containing 20 mM EDTA. The tissue/cell suspension was allowed to settle for 5 minutes before centrifuging and discarding the supernatant, and the cells were resuspended at 1 x 10^5^ cells/ml in ice-cold PBS. For blood samples, red blood cells were lysed with RBC lysis buffer, washed twice with ice-cold PBS, and then resuspended with ice-cold PBS. Individual cells were combined with low melting agarose at 37°C, embedded in agarose on slides and lysed with lysis buffer for 60 min. For DNA oxidative detection, cells were treated with and without Formamidopyrimidine DNA glycosylase (Fpg) (1:1000) (NEB, Cat # M0240S). Finally, the slides were electrophoresed under alkaline solution in a horizontal chamber to separate intact DNA from damaged fragments. Following electrophoresis, the samples were dried, stained with a DNA dye, and visualized by fluorescent microscopy (Andor Dragonfly spinning disk confocal, Oxford Instruments).

The comet assay fluorescent images were analyzed using *OpenComet* software (Gyori, Venkatachalam, Thiagarajan, Hsu, & Clement, 2014). The extent of DNA damage and oxidation were measured as a relative percentage of DNA in tail, normalized with Polg^wt/wt^ samples.

**γ****H2AX assay**

The γH2AX was detected in Mouse Embryo Fibroblasts (MEFs). MEFs were harvested from embryos from Polg^wt/mut^ female mice 14–15 days after the appearance of the copulation plug, as previously described (Durkin, Qian, Popescu, & Lowy, 2013). MEFs were cultured in high glucose DMEM (Gibco) with 10% fetal bovine serum (Gibco) and 1% Antibiotic-Antimycotic (Invitrogen). All cell lines were maintained at 37°C in a 5% CO_2_ incubator.

MEFs were seeded in 8-well chamber slides (Thermo Scientific) and fixed with 4% PFA in PBS for 10 min at room temperature (RT). After fixation, cells were washed three times with ice-cold PBS, and permeabilized with 0.5% Triton X-100 in PBS for 5 min at RT. Cells were then washed three times for 5 min with PBS, and blocked with Blocking Buffer (TBS, Invitrogen) for 2 hours at RT. Anti-γH2AX antibody (Invitrogen, CR55T33) was diluted to 1:200 with Blocking Buffer, and cells were incubated with the diluted antibody solution overnight at 4°C. The next day, MEFs were washed three times for 5 min with 0.1% TBST and incubated with Alexa Fluor 488 goat anti-mouse (Abcam, ab150113) diluted 1:200 with Blocking buffer for 2 hours at RT. After staining with the secondary antibody, MEFs were washed three times for 5 min with 0.1% TBST. The chambers were removed, and MEFs were embedded with Antifade Mounting Medium with DAPI (Vectashield). The Andor Dragonfly High Speed Confocal Microscope (Oxford Instruments) was used to take immunofluorescent images.

**Quantification of telomere length by real-time PCR**

Genomic DNA of blood samples was extracted with the DNeasy Blood & Tissue Kit (Qiagen, Cat # 69504). The absolute telomere length was detected by real-time PCR quantification assay kit (ScienCell, Cat # M8918), according to the manufacturer’s instructions.

**Quantification of telomere length by Southern Blot**

Genomic DNA was extracted from tissues using the QIAGEN Tip-100 Kit (Qiagen, Cat # 10223) according to the manufacturer’s instructions. Two antioxidant chemicals, butylated hydroxytoluene (Sigma, Product # PHR1117; DMSO solvent) and deferoxamine mesylate (Sigma, Product # D9533; Water solvent), were added to the G2 lysis buffer at a final concentration of 100 mM each to prevent accidental oxidation of the DNA during the DNA extraction process. The detection of telomere length and 8-oxoGua lesions in telomeric DNA were performed according to the previously described method (Fouquerel et al., 2019). To quantify the shortened telomeric DNA, boxes were drawn in each set of lanes to define the “bulk” and “tail” fractions (representing intact versus degraded telomeric DNA, respectively), and the signal for each fraction was quantified by Image J.

**Quantification of telomere length by Q-FISH**

Telomere length was measured using splenic lymphocytes. For the spleen preparation, the spleen was minced, and the lymphocytes cells were washed out of the spleen tissue using with RPMI1640 medium. The lymphocytes were then resuspended in RPMI1640 medium, placed in blood collection tubes, and mixed with TransFix stabilization solution (Cytomark, Cat # TFB-01-10) at a 1:5 ratio to prevent coagulation. The tubes were gently inverted ten times to ensure a thorough mixture, and then shipped to the cytology lab for processing.

To prepare cell for Q-FISH analysis, cells were spun down at 200 g for 10 min and re-suspended in 5 ml of 0.075 M KCl for 10 min at RT for 3D fixation of interphase nuclei, or for 30 min at RT for chromosome preparation. For 3D fixation, a hypotonic solution was overlaid with 1 ml of freshly prepared fixative (methanol/acetic acid, 3:1). For the preparation of chromosomes, a drop fixation method was used, as previously described (Beatty, Mai, & Squire, 2002). For the analysis of telomeres in interphase nuclei, cells were fixed in 3.7% formaldehyde/1× PBS for 10 min and washed three times in 1× PBS for 5 min. Incubation with 0.5% Triton X-100 for 10 min was used for removing the cell membrane and expose the cell nuclei. The slides were then equilibrated in 70% formamide/2X SSC pH 7.0 for 1 hour at RT before hybridization with 8 μl of PNA-telomere probe (DAKO, Glostrup, Denmark). The Hybrite^TM^ (Vysis/Abbott) was used for the hybridization, with the denaturation step set at 3 min 80°C, and the hybridization step set at 2 hours at 30°C. DAPI was used as a counterstain for the DNA. The mounting medium Vectashield (Vector Laboratories, Burlingame, CA, Cat # H-1200-10) was used to prevent photobleaching of the sample.

Image acquisition was performed on 30+ interphase nuclei per cell line using an Axio Imager Z2 microscope (Carl Zeiss, Inc. Canada) and an AxioCam MRm (Carl Zeiss, Inc. Canada). A 63/1.4 oil objective lens was used for all images. The TeloView software (Vermolen et al., 2005) (Telo Genomics, Toronto, ON, Canada) was used to determine telomeric signal intensity (telomere length), the average telomere intensity, the number of telomeric signals, the number of telomere aggregates, and the *a/c* ratio (Mathur et al., 2014).

**Quantification of total mtDNAcn and linear mtDNAcn by real-time PCR**

Total DNA was extracted using the DNeasy Blood & Tissue Kit (Qiagen, Cat # 69504) according to the manufacturer’s instructions. 80 ng DNA of each sample was digested by 2 units of Exonuclease V in a total volume of 40 µl reaction solution (NEB, Cat # M0345S), followed by incubation at 37°C for 30 min and 65°C for 30 min. The DNA concentration was quantified with the Qubit dsDNA HS Assay kit (Invitrogen, REF # Q32854).

The total mtDNAcn and linear mtDNAcn were measured by a quantitative PCR-based method using the CFX96 Real-Time PCR System (Bio-Rad Laboratories). The mtDNAcn was quantified as the ratio of a mitochondrial gene copy number (tRNA-Val) to a single-copy nuclear gene (β2-Microglobulin, B2M), using previously published and validated primers (Ishimoto et al., 2017). The tRNA-Val primers specifically amplify a sequencing that is not duplicated in the nuclear genome as nuclear mitochondrial insertion (NUMT). The primers sequences were as follows: tRNA-Val- forward 5’-CTAGAAACCCCGAAACCAAA-3’ and tRNA-Val- reverse 5’-CCAGCTATCACCAAGCTCGT-3’; B2M- forward 5’-ATGGGAAGCCGAACATACTG-3’ and B2M- reverse 5’-CAGTCTCAGTGGGGGTGAAT-3’. The 20 μl PCR reaction solution contained 1X SYBR Green Master Mix (Qiagen, Cat # 204143), 500 nM of each primer, and 40 ng of total DNA. Real-time PCR conditions were 95°C for 10 min; 95°C for 20 s, 58°C for 20 s and 72°C for 45 s, 45 cycles; 72°C for 10 min.

For linear mtDNAcn, the average Ct value of Exonuclease V treated samples was set as the Ct treated. ΔCt was calculated as the untreated Ct value minus treated Ct value. The relative amount of untreated DNA as compared to the treated DNA level (RE) was calculated using the comparative quantification method, where RE=2^-ΔCt^. The following formula was used to calculate the percentage of linear mtDNAcn: (RE-1)/RE*100%, normalizing with Polg^wt/wt^.

**Quantification of 8-oxodG levels in mtDNA and telomeric DNA by real-time PCR**

DNA samples were incubated with Fpg enzyme according to a previously published protocol, with some modifications (O'Callaghan, Baack, Sharif, & Fenech, 2011). Briefly, 40 ng DNA of each sample was digested by 2.56 units of Fpg enzyme in a total volume of 20 µl reaction solution (NEB, Cat # M0240S), followed by incubation at 37°C for 120 min and 65°C for 20 min. The control group replaced Fpg with water, with all other conditions being the same. The DNA concentration was quantified with the Qubit dsDNA HS Assay kit (Invitrogen, REF # Q32854).

The 8-oxodG levels in mtDNA and telomeric DNA were measured by a quantitative PCR-based method using the CFX96 Real-Time PCR System (Bio-Rad Laboratories). The 8-oxodG level in mtDNA was quantified as the ratio of a mitochondrial gene copy number (tRNA-Val), with or without Fpg treatment. The 8-oxodG level in telomeric DNA was quantified as the ratio of the real-time PCR-detected Telomere levels, with or without Fpg treatment.

Primer sequences were as follows: tRNA-Val- forward 5’- CTAGAAACCCCGAAACCAAA-3’ and tRNA-Val- reverse 5’-CCAGCTATCACCAAGCTCGT-3’; Telo- forward 5’-CGGTTTGTTTGGGTTTGGGTTTGGGTTTGGGTTTGGGTT-3’ and Telo- reverse 5’-GGCTTGCCTTACCCTTACCCTTACCCTTACCCTTACCCT-3’. The 20 μl PCR reaction solution contained 1X SYBR Green Master Mix (Qiagen, Cat # 204143), 1-2 ng of total DNA, 100 nM of each primer for mtDNA and telomeric DNA. Real-time PCR conditions of mtDNA were 95°C for 10 min; 95°C for 30 s, 58.3°C for 60 s, 45 cycles; 72°C for 10 min. Real-time PCR conditions for telomeric DNA were 95°C for 10 min; 95°C for 30 s, 56.3°C for 60 s, 45 cycles; 72°C for 10 min. The following formula was used to calculate the change in cycle threshold: ΔCt= Ct treated-Ct untreated (O'Callaghan et al., 2011).

**References**

Arneson, A., Haghani, A., Thompson, M. J., Pellegrini, M., Kwon, S. B., Vu, H., . . . Barnes, B. (2021). A mammalian methylation array for profiling methylation levels at conserved sequences. *bioRxiv*. doi:10.1101/2021.01.07.425637

Beatty, B., Mai, S., & Squire, J. (2002). *FISH: a practical approach*: Oxford University Press.

Coninx, E., Chew, Y. C., Yang, X., Guo, W., Coolkens, A., Baatout, S., . . . Quintens, R. (2020). Hippocampal and cortical tissue-specific epigenetic clocks indicate an increased epigenetic age in a mouse model for Alzheimer’s disease. *Aging (Albany NY), 12*(20), 20817. doi:10.18632/aging.104056

Durkin, M. E., Qian, X., Popescu, N. C., & Lowy, D. R. (2013). Isolation of mouse embryo fibroblasts. *Bio-protocol, 3*(18), e908-e908. doi:10.21769/BioProtoc.908

Fouquerel, E., Barnes, R. P., Uttam, S., Watkins, S. C., Bruchez, M. P., & Opresko, P. L. (2019). Targeted and Persistent 8-Oxoguanine Base Damage at Telomeres Promotes Telomere Loss and Crisis. *Molecular cell, 75*(1), 117-130 e116. doi:10.1016/j.molcel.2019.04.024

Gyori, B. M., Venkatachalam, G., Thiagarajan, P., Hsu, D., & Clement, M.-V. (2014). OpenComet: an automated tool for comet assay image analysis. *Redox Biology, 2*, 457-465. doi:10.1016/j.redox.2013.12.020

Horvath, S. (2013). DNA methylation age of human tissues and cell types. *Genome biology, 14*(10), 1-20. doi:10.1186/s13059-015-0649-6

Horvath, S., Langfelder, P., Kwak, S., Aaronson, J., Rosinski, J., Vogt, T. F., . . . Waldvogel, H. J. (2016). Huntington's disease accelerates epigenetic aging of human brain and disrupts DNA methylation levels. *Aging (Albany NY), 8*(7), 1485. doi:10.18632/aging.101005

Horvath, S., & Levine, A. J. (2015). HIV-1 infection accelerates age according to the epigenetic clock. *The Journal of infectious diseases, 212*(10), 1563-1573. doi:10.1093/infdis/jiv277

Ishimoto, Y., Inagi, R., Yoshihara, D., Kugita, M., Nagao, S., Shimizu, A., . . . Zhou, J. (2017). Mitochondrial abnormality facilitates cyst formation in autosomal dominant polycystic kidney disease. *Molecular cellular biology, 37*(24), e00337-00317. doi:10.1128/MCB.00337-17

Mathur, S., Glogowska, A., McAvoy, E., Righolt, C., Rutherford, J., Willing, C., . . . Garcia, A. (2014). Three-dimensional quantitative imaging of telomeres in buccal cells identifies mild, moderate, and severe Alzheimer's disease patients. *Journal of Alzheimer's Disease, 39*(1), 35-48. doi:10.3233/JAD-130866

Mozhui, K., Lu, A. T., Li, C. Z., Haghani, A., Sandoval-Sierra, J. V., Williams, R. W., & Horvath, S. (2021). Genetic Analyses of Epigenetic Predictors that Estimate Aging, Metabolic Traits, and Lifespan. *bioRxiv*. doi:10.1101/2021.06.23.449634

Nie, B., Gan, W., Shi, F., Hu, G.-X., Chen, L.-G., Hayakawa, H., . . . Cai, J.-P. (2013). Age-dependent accumulation of 8-oxoguanine in the DNA and RNA in various rat tissues. *Oxidative medicine cellular longevity, 2013*. doi:10.1155/2013/303181

O'Callaghan, N., Baack, N., Sharif, R., & Fenech, M. (2011). A qPCR-based assay to quantify oxidized guanine and other FPG-sensitive base lesions within telomeric DNA. *Biotechniques, 51*(6), 403-412. doi:10.2144/000113788

Vermolen, B., Garini, Y., Mai, S., Mougey, V., Fest, T., Chuang, T. Y., . . . Young, I. (2005). Characterizing the three‐dimensional organization of telomeres. *Cytometry Part A: the journal of the International Society for Analytical Cytology, 67*(2), 144-150. doi:10.1002/cyto.a.20159

Zhou, W., Triche Jr, T. J., Laird, P. W., & Shen, H. (2018). SeSAMe: reducing artifactual detection of DNA methylation by Infinium BeadChips in genomic deletions. *Nucleic Acids Research, 46*(20), e123-e123. doi:10.1093/nar/gky691
